# Amelioration of intestinal barrier function and reduction of blood lead level in adult women with recurrent spontaneous abortion by a novel product of dietary fiber mixture, Holofood

**DOI:** 10.1186/s41043-023-00394-2

**Published:** 2023-07-07

**Authors:** Ye Tian, Zhiyuan Pan, Liling Lan, Yuxiao Chang, Ting Zhao, Zhihong Fu, Shuhua Wu, Tianqin Deng, Meilan Cao, Weizhou Wang, Yujing Bi, Ruifu Yang, B. J. Yang Lee, Qingzhi Liu

**Affiliations:** 1 Reproductive Medicine Center, Department of Obstetrics and Gynecology, Chinese People’s Liberation Army General Hospital, Beijing, 100853 China; 2 Shenzhen Key Laboratory of Fertility Regulation, Center of Assisted Reproduction and Embryology, The University of Hong Kong - Shenzhen Hospital, Shenzhen, 518053 China; 3 State Key Laboratory of Pathogen and Biosecurity, Beijing Institute of Microbiology and Epidemiology, Beijing, 100071 China; 4 Affiliated Shenzhen Maternity & Child Healthcare Hospital, Southern Medical University, No. 3012, Fuqiang Road, Futian District, Shenzhen, 518028 China; 5 Guangzhou First People’s Hospital, Guangzhou, 510180 China; 6 Center of Assisted Reproduction and Embryology, The University of Hong Kong - Shenzhen Hospital, Shenzhen, 518053 China; 7 Department of Obstetrics and Gynecology, The Seventh Medical Center of Chinese People’s Liberation Army General Hospital, Beijing, 100007 China; 8 Beijing Future Science & Technology Development Co., Ltd., Rm. 1702A #1 Guanhu International Plaza, 105 Yaojiayuan Road, Chaoyang District, Beijing, 100025 China

**Keywords:** Recurrent spontaneous abortion, Intestinal barrier integrity, Lead, Dietary fibers, d-lactate, Diamine oxidase

## Abstract

**Background:**

The elevated circulating toxins secondary to the impairment of intestinal barrier integrity commonly elicit a chronic inflammatory response and finally contribute to multiple diseases. These toxins, including bacterial by-products and heavy metals, are the potent risk factors for the development of recurrent spontaneous abortion (RSA). Preclinical evidence suggests that several dietary fibers can restore intestinal barrier function and decrease the accumulation of heavy metals. However, it is uncertain whether treatment with a newly developed blend of dietary fibers product (Holofood) benefits patients with RSA.

**Methods:**

In this trial, we enrolled 70 adult women with RSA, who were randomly assigned into the experiment group and the control group in a 2:1 ratio. Upon the basis of conventional therapy, subjects in the experiment group (*n* = 48) received 8 weeks oral administration with Holofood three times daily at a dose of 10 g each time. Subjects without Holofood consumption were set as the control (*n* = 22). Blood samples were collected for the determinations of metabolic parameters, heavy mental lead, and the indices related to intestinal barrier integrity (d-lactate, bacterial endotoxin, and diamine oxidase activity).

**Results:**

The reduction amplitude in blood lead from baseline to week 8 was 40.50 ± 54.28 (μg/L) in the experiment group as compared with 13.35 ± 36.81 (μg/L) in the control group (*P* = 0.037). The decreased level of serum d-lactate from baseline to week 8 was 5.58 ± 6.09 (mg/L) in the experiment group as compared with − 2.38 ± 8.90 (mg/L, *P* < 0.0001) in the control group. The change in serum DAO activity from baseline to week 8 was 3.26 ± 2.23 (U/L) in the experiment group as compared with − 1.24 ± 2.22 (U/L, *P* < 0.0001) in the control group. Participants who received Holofood had a greater decline in blood endotoxin from baseline to week 8 than those in the control group. Moreover, by comparing with the self-baseline, Holofood consumption significantly decreased the blood levels of lead, d-lactate, bacterial endotoxin, and DAO activity.

**Conclusion:**

Our results suggest that Holofood affords a clinically relevant improvements in blood lead level and intestinal barrier dysfunction in patients with RSA.

## Introduction

Approximately 2.5% of women attempting to conceive suffer from recurrent spontaneous abortion (RSA) [1]. This is one of the major pregnancy disorders that severely threatens the physical, mental, and reproductive health of patients [2]. Although the etiology of 50% RSA cases is still unknown [3], several risk factors have been proven to account for such intractable complication, containing gene [4], anatomy [5], endocrine [6], immune [7], environmental pollution [8], and heavy metals exposure [9]. For example, it has been recognized that pregnancy must induce immune tolerance to avoid fetal rejection, while obesity can cause chronic inflammation through activating the immune system. This impaired maternal immuno-tolerance will lead to pregnancy failure such as RSA, one of the most common complications during early pregnancy [10].

Accumulating evidence suggests that lead is one of the most toxic heavy metals prevalent in the contaminated environment [11]. After absorption, lead would impair physiological functions in multiple organs [12, 13]. The raised blood lead level is well associated with higher risk of pregnancy loss [14], since lead could directly damage female reproductive function via inducing menstrual abnormalities, spontaneous abortion, and premature delivery [15].

Given that gut microbiota plays a critical role in the maintenance of intestinal homeostasis [16], there is a growing interest in exploring the interactions between lead toxicity and gut microbiota in rodents and humans [17]. Recently, a hypothesis has been proposed that lead exposure greatly influences the gut microbiota, leading to an impaired gut barrier integrity and an increased intestinal permeability [18], which facilitates toxins to enter the enterohepatic circulation and finally results in many chronic inflammatory diseases [19]. In this respect, preclinical evidence suggested that lead exposure not only altered the composition of gut microbial community, but also impacted metabolic functions, leading to gut dysbiosis in mice [20]. Modulation of the gut microbiota by probiotics [21] and prebiotic galacto-oligosaccharide (GOS) [22], or oral supplementation of lead-intolerant intestinal microbes [23], all provided significant protections against lead toxicity in mice. The underlying mechanism involves in biotransformation of lead, such as prevention of absorption [21], promotion of fecal excretion, reduction of blood concentration [23], as well as recovery of the gut barrier integrity and functions [22]. Intriguingly, recent clinical outcomes conducted in pregnant women and children showed that probiotic-supplemented yogurt possessed similar beneficial effects to reduce the bioaccumulation of heavy metal arsenic and mercury [24]. Accordingly, targeting gut microbiota with probiotics, prebiotics, or dietary fibers appears to be a promising approach to ameliorating intestinal barrier function and decreasing the levels of heavy metals [18, 25, 26].

Holofood, a newly developed product of dietary fibers mixture with unique nutritionally balanced ratio, has been previously displayed to upregulate the gut microbial diversity and decrease the activity of serum diamine oxidase (DAO), which eventually helps to maintain the health of the intestinal microecosystem [26]. Furthermore, Holofood was reported to alleviate hypoxia-induced cardiac hypertrophy in rats via modulating the gut microbiome and metabolic profiles [27]. Nevertheless, it is still uncertain whether consumption of such dietary fibers blend benefits patients with RSA.

Therefore, we conducted a randomized open-label trial to investigate the effects of Holofood treatment for 8 weeks on adult women with RSA, and focused on evaluating the changes of metabolic parameters, blood lead levels, and the indices referred to intestinal barrier integrity in participants. Determinations of bacterial by-products endotoxin and d-lactate were used to reflect the intestinal permeability, while test of DAO activity, an enzyme that released from the intestinal epithelia, was used to reflect the degree of gut barrier impairment [28].

## Methods

### Trial design

From November 2019 to November 2020, this randomized, open-label, parallel-group controlled trial was conducted in accordance with the principles of the Declaration of Helsinki and Good Clinical Practice guidelines, with the clinical trial registration number of ChiCTR1900026336. The present protocol was approved by the Ethics Committee for Clinical Research of the hospital with the number of SFYLS-2020-011. All recruited participants were informed the related risks and complications and signed an informed consent form. Investigators were responsible for data collection and analysis.

### Participants

According to the RSA diagnostic criteria, adult women aged 22 to 42 years who experienced two or more miscarriages or three or more consecutive cycles of high-quality embryo transfer without pregnancy were recruited, with blood lead concentration > 20 µg/l. In this populations, patients with immune dysfunction and abnormalities of glucose or lipid metabolisms were also enrolled. Key exclusion criteria were: (1) Un-heathy life styles such as smoking and drinking. (2) Excessive exposure to harmful chemicals. (3) Genetic problems such as chromosomal abnormalities. (4) Uterine anatomy abnormalities such as a mediastinal uterus and cervical insufficiency. (5) Abnormal endocrine function such as thyroid disease and hyperprolactinemia. (6) Reproductive tract infections. (7) Use of antibiotics within 90 days before enrollment. (8) The habits of taking biotic products such as probiotics, prebiotics, synbiotics, or postbiotics. (9) A history of administrations with hypoglycemia and hypolipidemic drugs during the past two months. (10) The changed amplitude of body weight over 5% in the past two months. Patients with the coexisting conditions (i.e., severe myocardial ischemia, proliferative retinopathy, renal insufficiency with the proteinuria > 1 g/day, hypertension with systolic pressure > 160 mmHg or diastolic blood pressure > 95 mmHg) were also excluded.

### Procedures

**Fig. 1:**
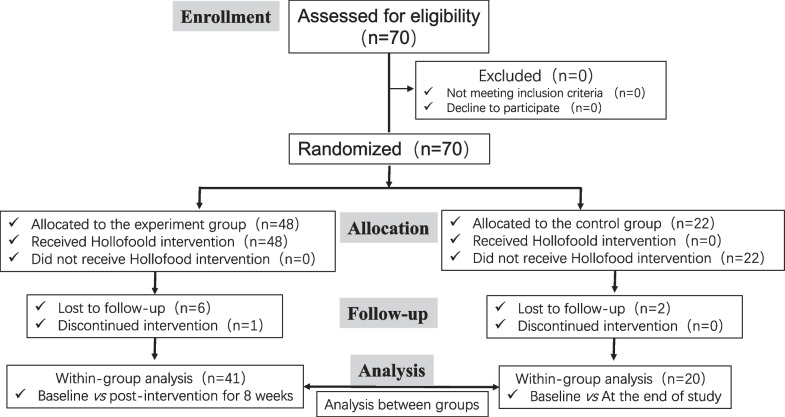
CONSORT flow diagram of the participants in this trial

The age, weight, and body mass index (BMI) in participants were recorded in detail, together with their history of gravidity, miscarriage, and implantation failure. A total of 70 eligible subjects were randomly assigned in a 2:1 ratio to the experimental group and the control group. Simple randomization strategy was performed for grouping. Random numbers generated using SPSS software (version 22.0) were sorted in ascending order, then those with numbers from 1 to 48 were assigned to the experiment group, while those with numbers from 49 to 70 were assigned to the control group. Upon the basis of the conventional therapy with multivitamins (take one pill daily with a meal), subjects in the experiment group (*n* = 48) received 8 weeks oral administration with Holofood three times daily at a dose of 10 g each time (taken 30 min before each meal), whereas subjects without Holofood consumption (*n* = 22) were set as control. Of the 70 participants, finally the data of 41 subjects in the experiment group and 20 subjects in the control group were included into the outcomes analysis. The CONSORT flow diagram of the participants in this trial was illustrated in Fig. 1.

Holofood, a blend consisting of four kinds of prebiotics (galacto-oligosaccharides (GOS), inulin, yeast β-glucan, polydextrose) and four different plant-derived soluble dietary fibers (microcrystalline cellulose, erythritol, guar gum, steviol glycosides), was provided by Beijing Ruiqianjing Technology Development Co., Ltd., with the production license number of SC10611151014507.

### Blood lead quantification

Graphite furnace atomic absorption spectrometry was applied to determine the levels of blood lead. Briefly [29], a 100-µL fresh whole-blood sample was digested with 1 ml 0.5% Triton X-100, followed by adding 100 µL 1% nitric acid to decompose the high organic content of the matrix, and then analyzed by a BOHUI 2100 analyzer (Beijing Bohui Innovation Technology Co., Ltd., Beijing, China), according to the standard operating procedure. The electrodeless discharge lamp was operated at 10 mA current, and spectral line at 283.8 nm wavelength was selected for the determination with a spectral bandpass of 0.5 nm.

### Evaluation of metabolic parameters

About 5 ml of fasting venous blood sample was collected from each subject before and after 8 weeks Holofood intervention using blood collection vessels containing procoagulants. Serum was extracted after centrifuging, and the levels of uric acid, total cholesterol, triglyceride, high-density lipoprotein, low-density lipoprotein, and glycosylated hemoglobin were measured at Shenzhen Maternal and Child Health Hospital.

#### Determinations of indices related to intestinal barrier integrity

Serum samples isolated from fresh whole-blood were snap stored at − 80 °C. After all samples were collected, the levels of DAO activity, d-lactate, and bacterial endotoxin were quantitatively tested by the JY-DLT intestinal barrier function biochemical index analysis system with the matching joint detection kit (enzymatic method, JY-Po-Color-DLT set, Beijing Zhongjin Golden Field) [30].

### Statistical analysis

The data were expressed as mean ± SD and analyzed by the SPSS software package (version 24.0). Demographic and clinical characteristics of the participants at baseline between groups were analyzed by the independent samples *t*-test, except for the comparison of delivery times with the Chi-square test. Comparisons of key metrics within the experiment group between pre- and post-intervention were performed by paired *t*-test. The mean substitution method was used to impute the missing data. *P* < 0.05 indicated a statistically significant difference.

## Results

### Baseline characteristics of participants

No significant differences in age, weight, BMI, gravidity, and history of adverse pregnancy or implantation failure were observed between the two groups, showing the similar demographics and baseline characteristics (Table 1).

**Table 1:** Demographic and clinical characteristics of the participants at baseline

Characteristic	Holofood (*n* = 48)	Control (*n* = 20)	*p* value
Age(year)	32.71 ± 4.06	30.58 ± 5.25	0.068
Weight (kg)	56.60 ± 5.74	54.49 ± 6.99	0.189
BMI (kg/m^2^)	22.34 ± 2.18	21.30 ± 2.33	0.076
History of production number	40/48(83%)	15/22(68%)	0.210
Gravidity (time)	2.71 ± 1.50	3.00 ± 1.82	0.484
History of adverse pregnancy or replantation failure(time)	2.08 ± 1.10	1.79 ± 1.33	0.337

BMI: The body mass index is the weight in kilograms divided by the square of the height in meters. Excepting for the comparison of history of production number performed by the Chi-square test, other statistics were performed by independent samples *t* test

### Holofood did not affect metabolic parameters in participants

There were no significant changes in blood uric acid, total cholesterol, triglycerides, high-density lipoprotein, low-density lipoprotein, and glycosylated hemoglobin levels between groups, either at baseline or at the end of study. In experiment group, by comparing with self-baseline, Holofood intervention for 8 weeks had no significant effects on these parameters, indicating that Holofood did not affect the basic metabolism homeostasis (Table 2).

**Table 2:** Within-group comparisons of metabolic parameters in the experiment group by paired *t* test

Parameters	Baseline	After Holofood intervention	Mean difference	95% Confidence interval	*p* value
URIC (μmol/L)	276.98 ± 75.40	271.27 ± 54.57	− 4.373	− 34.523 to 25.774	0.741
TC (mmol/L)	4.79 ± 1.34	4.65 ± 1.00	0.162	− 0.372 to 0.693	0.555
TG (mg/L)	1.16 ± 0.66	1.16 ± 0.75	0.003	− 0.303 to 0.312	0.990
HDL (mmol/L)	1.46 ± 0.38	1.44 ± 0.36	− 0.008	− 0.177 to 0.161	0.738
LDL (mmol/L)	2.79 ± 1.20	2.62 ± 0.96	0.121	− 0.369 to 0.612	0.323
HbA1c (mmol/L)	5.20 ± 0.43	5.08 ± 0.44	0.147	− 0.057 to 0.350	0.055

URIC: Uric acid; TC: Total cholesterol; TG: Triglyceride; HDL: High-density lipoprotein; LDL: Low-density lipoprotein; HbA1c: Glycosylated hemoglobin

### Holofood improved the intestinal mucosal barrier in patients with RSA

In the experimental group, statistically significant reductions in blood DAO activity, d-lactate, and bacterial endotoxin were observed after intervention with Holofood (Table 3).

**Table 3:** Within-group comparisons of blood lead and intestinal barrier integrity indices in the experiment group by paired *t*-test

Parameters	Baseline	After Holofood intervention	Mean difference	95% Confidence interval	*p* value
Lead (μg/L)	75.81 ± 64.34	33.61 ± 21.67	41.650	22.318 to 60.982	0.000
DAO activity (U/L)	10.81 ± 2.43	7.58 ± 1.33	3.231	2.359 to 4.105	0.000
d-lactate (mg/L)	15.76 ± 6.37	10.22 ± 3.84	5.539	3.194 to7.883	0.000
Endotoxin (U/L)	10.65 ± 0.76	10.39 ± 0.16	0.265	0.020 to 0.511	0.040

DAO: Diamine oxidase

We noted that in the control group, there were significantly increased levels of DAO activity and d-lactate after the conventional therapy with multivitamins (Table 4). To identify the benefits of Holofood, we calculated the changed values of these indices in each subject from baseline to week 8 and further compared their differences between groups. As shown in Table 5, from baseline to week 8, the decreased amplitudes of serum d-lactate and DAO activity in the experiment group were both significantly greater than those in the control group. Moreover, despite no statistical significance, participants who received Holofood intervention had a relatively greater decline of blood bacterial endotoxin from baseline to week 8 than those who in the control group. These results indicated that Holofood indeed improved the intestinal barrier function in patients with RSA.

**Table 4:** Within-group comparisons of blood lead and intestinal barrier integrity indices in the control group by paired *t*-test

Parameters	Baseline	After 8 weeks	Mean difference	95% Confidence interval	*p* value
Lead (μg/L)	50.46 ± 27.01	43.49 ± 23.77	17.174	− 3.813 to 38.162	0.287
DAO activity (U/L)	7.72 ± 1.78	9.11 ± 2.81	− 1.032	− 2.551 to 0.488	0.023
d-lactate (mg/L)	10.82 ± 5.02	13.96 ± 9.38	− 1.839	− 6.429 to 2.751	0.017
Endotoxin (U/L)	9.80 ± 1.59	10.31 ± 0.93	− 0.051	− 1.339 to 1.238	0.211

DAO: Diamine oxidase

**Table 5:** Changed amplitude of blood lead and intestinal barrier integrity indices between groups by independent samples *t*-test

Parameters	Control	Experiment (Holofood intervention)	Mean difference	95% Confidence interval	*p* value
Lead (μg/L)	13.35 ± 36.81	40.50 ± 54.28	13.958	7.578 to 63.502	0.037
DAO activity (U/L)	− 1.24 ± 2.22	3.26 ± 2.23	0.695	3.063 to 5.846	0.000
d-lactate (mg/L)	− 2.38 ± 8.90	5.58 ± 6.09	8.180	3.897 to 12.463	0.000
Endotoxin (U/L)	− 0.03 ± 2.45	0.26 ± 0.72	0.786	0.144 to 1.428	0.584

DAO: Diamine oxidase

### Holofood decreased the level of blood lead in patients with RSA

From baseline to week 8, a remarkable reduction of blood lead level was found between pre- and post-Holofood intervention in the experiment group (Table 3), while no obvious change of that was observed in the control group (Table 4). Furthermore, the magnitude of decline in blood lead from baseline to week 8 in the experiment group was substantially stronger than that in the control group (Table 5).

## Discussion

In recent decades, it has been well recognized that environmental pollution increases the risk of human exposure to heavy metal lead, which may consequently produce chronic or acute accumulation, and finally results in impairments of female and male reproductive system [31]. Indeed, a prospective clinical outcome showed that there was a striking dose–response relationship between blood lead and risk of spontaneous abortion [32], and even a small quantity of blood lead was harmful [33]. Because exposure to a very low level of lead significantly modified placental Ca^2+^ transport in pregnant women [34]. Notably, in this trial, we found that adult women with RSA who experienced two or more miscarriages or three or more consecutive cycles of embryo transfer without pregnancy had a marked reduction in blood lead level from baseline with Holofood as an adjunct to conventional intervention. Such reduction of blood lead that we observed in the Holofood intervention group is substantially exceeded that in the control group without Holofood treatment, suggesting a clinically meaningful response in patients with RSA.

Theoretically, prebiotics and dietary fibers in the Holofood are indigestible food ingredients which can serve as the substrates for the fermentation by several gut microbes, especially for the promotion of probiotics growth, and therefore help to maintain intestinal homeostasis [35]. Considering the major effector site of Holofood is the gut, together with the fact that impairment of intestinal barrier integrity with increased permeability is one of the lead toxicities [17, 20], it is very likely that Holofood decreases the level of blood lead because of its improvements on the gut barrier function. As expected, Holofood administration for 8 weeks in patients with RAS significantly decreased the circulating d-lactate and DAO activity, the two well-known intestinal hyper permeability biomarkers [36]. Recently, dietary fibers are increasingly recognized to play many beneficial roles in gut health [37]. Several clinical trials also identified that dietary fiber has the potential to change the gut microbiota and alter metabolic regulation [38]. Furthermore, prebiotics was reported to restore a degraded mechanical barrier in an animal study of anti-inflammatory nutritional food mechanisms [39].

One of the components in Holofood product is galacto-oligosaccharides (GOS), which can be selectively used by probiotics, such as *Bifidobacterium* and *Lactobacillus*, while inhibiting the proliferation of gram-negative pathogenic bacteria and maintaining the balance of gut microbiota [40–42]. Importantly, modulation of the gut microbiota by GOS was recently found to promote fecal lead excretion and reduces lead accumulation in the blood and tissues in mice, since GOS supplementation enhanced the abundance of bacteria with good lead-binding ability and restored the intestinal barrier function [22]. Another key ingredient in Holofood product is inulin, a famous prebiotic which also can promote the abundance of *Bifidobacterium* [43]. Dietary inulin supplementation was showed to decrease DAO activity, elevated the *Lactobacillus* population but decreased the *Escherichia coli* population in a porcine model [44]. The dietary inulin also offered a promising approach to avoid post-weaning gastrointestinal tract disorders in pigs [45]. These findings, together with our results, suggested that prebiotics intervention has the potential to protect against intestinal barrier dysfunction.

In the current study, we found that Holofood product did not change the circulating metabolic parameters including uric acid, high-density lipoprotein, low-density lipoprotein, and glycosylated hemoglobin, indicating that the Holofood product did not affect the normal metabolic homeostasis of the body. In contrast, GOS supplementation could improve blood glucose and lipid metabolism because daily supplementation of GOS for 3 months reduced total cholesterol levels in women by 13% compared with the baseline [46]. Our results showed that total cholesterol had a downward trend but no statistical difference after intervention with Holofood including GOS. One explanation is that the average BMI of the patients in this study was less than 24, and blood glucose and lipid metabolism were also within the physiological ranges but not in the pathophysiological state. In this study, we showed that intervention with a novel dietary fibers blend product Holofood affords a clinically relevant improvements in blood lead level and intestinal barrier function in patients with RSA.

### Limitations

Three major limitations exist in the present study. First, although it has been enough to conclude the clinical improvements after Holofood consumption through using paired *t* test and independent samples *t* test to analyze the differences within-group and between group, we still acknowledged that using ANCOVA test may be able to obtain a higher statistical efficiency. Second, we did not examine the corresponding alteration of gut microbiota that has been conformed to commonly mediate the improvement of intestinal barrier function by prebiotics. Investigating the changes in gut microbiota may be able to explain the effect of Holofood on blood lead level in RSA. Third, we did not observe the effect of Holofood on the pregnancy outcome. Similarly, growth hormone supplementation has been shown to improve oocyte quality and live birth, but few studies have examined whether growth hormone can reduce embryonic aneuploidy [47]. The effect of Holofood on pregnancy outcome requires rigorous clinical trial validation.

## Data Availability

The data that support the findings of this study are available from the corresponding author upon reasonable request.

## Abbreviations

RSA: Recurrent spontaneous abortion
DAO: Diamine oxidase activity
GOS: Galacto-oligosaccharide
BMI: Body mass index
SPSS: Statistical product and service solutions

## References

[1] Dimitriadis E, Menkhorst E, Saito S, Kutteh WH, Brosens JJ (2020). Recurrent pregnancy loss. Nat Rev Dis Primers.

[2] Quenby S, Gallos ID, Dhillon-Smith RK, Podesek M, Stephenson MD, Fisher J, Brosens JJ, Brewin J, Ramhorst R, Lucas ES (2021). Miscarriage matters: the epidemiological, physical, psychological, and economic costs of early pregnancy loss. Lancet.

[3] Garrido-Gimenez C, Alijotas-Reig J (2015). Recurrent miscarriage: causes, evaluation and management. Postgrad Med J.

[4] Tise CG, Byers HM (2021). Genetics of recurrent pregnancy loss: a review. Curr Opin Obstet Gynecol.

[5] Turocy JM, Rackow BW (2019). Uterine factor in recurrent pregnancy loss. Semin Perinatol.

[6] Amrane S, McConnell R (2019). Endocrine causes of recurrent pregnancy loss. Semin Perinatol.

[7] Li D, Zheng L, Zhao D, Xu Y, Wang Y (2021). The Role of Immune Cells in Recurrent Spontaneous Abortion. Reprod Sci.

[8] Canipari R, De Santis L, Cecconi S (2020). Female fertility and environmental pollution. Int J Environ Res Public Health.

[9] Dutta S, Gorain B, Choudhury H, Roychoudhury S, Sengupta P (2022). Environmental and occupational exposure of metals and female reproductive health. Environ Sci Pollut Res Int.

[10] Li Y, Chen J, Lin Y, Xu L, Sang Y, Li D, Du M (2021). Obesity challenge drives distinct maternal immune response changes in normal pregnant and abortion-prone mouse models. Front Immunol.

[11] Wani AL, Ara A, Usmani JA (2015). Lead toxicity: a review. Interdiscip Toxicol.

[12] Yadav G, Chambial S, Agrawal N, Gothwal M, Kathuria P, Singh P, Sharma P, Sharma PP (2020). Blood lead levels in antenatal women and its association with iron deficiency anemia and adverse pregnancy outcomes. J Family Med Prim Care.

[13] Matovic V, Buha A, Ethukic-Cosic D, Bulat Z (2015). Insight into the oxidative stress induced by lead and/or cadmium in blood, liver and kidneys. Food Chem Toxicol.

[14] Kaur M, Sharma P, Kaur R, Khetarpal P (2022). Increased incidence of spontaneous abortions on exposure to cadmium and lead: a systematic review and meta-analysis. Gynecol Endocrinol.

[15] Zhou Y, Yan L, Li H, Li X, Liu Y, Liu J (2021). Patterns and determinants of essential and toxic elements in Chinese women at mid-pregnancy, late pregnancy, and lactation. Nutrients.

[16] Fan Y, Pedersen O (2020). Gut microbiota in human metabolic health and disease. Nat Rev Microbiol.

[17] Assefa S, Kohler G (2020). Intestinal microbiome and metal toxicity. Curr Opin Toxicol.

[18] Yu L, Yu Y, Yin R, Duan H, Qu D, Tian F, Narbad A, Chen W, Zhai Q (2021). Dose-dependent effects of lead induced gut injuries: an in vitro and in vivo study. Chemosphere.

[19] Liu W, Feng H, Zheng S, Xu S, Massey IY, Zhang C, Wang X, Yang F (2021). Pb toxicity on gut physiology and microbiota. Front Physiol.

[20] Gao B, Chi L, Mahbub R, Bian X, Tu P, Ru H, Lu K (2017). Multi-omics reveals that lead exposure disturbs gut microbiome development, key metabolites, and metabolic pathways. Chem Res Toxicol.

[21] Zhai Q, Liu Y, Wang C, Qu D, Zhao J, Zhang H, Tian F, Chen W (2019). Lactobacillus plantarum CCFM8661 modulates bile acid enterohepatic circulation and increases lead excretion in mice. Food Funct.

[22] Zhai Q, Wang J, Cen S, Zhao J, Zhang H, Tian F, Chen W (2019). Modulation of the gut microbiota by a galactooligosaccharide protects against heavy metal lead accumulation in mice. Food Funct.

[23] Zhai Q, Qu D, Feng S, Yu Y, Yu L, Tian F, Zhao J, Zhang H, Chen W (2019). Oral supplementation of lead-intolerant intestinal microbes protects against lead (Pb) toxicity in mice. Front Microbiol.

[24] Bisanz JE, Enos MK, Mwanga JR, Changalucha J, Burton JP, Gloor GB, Reid G (2014). Randomized open-label pilot study of the influence of probiotics and the gut microbiome on toxic metal levels in Tanzanian pregnant women and school children. mBio.

[25] Feng P, Ye Z, Kakade A, Virk AK, Li X, Liu P (2018). A review on gut remediation of selected environmental contaminants: possible roles of probiotics and gut microbiota. Nutrients.

[26] Yao M, Shao X, Wei Y, Zhang X, Wang H, Xu F (2022). Dietary fiber ameliorates lead-induced gut microbiota disturbance and alleviates neuroinflammation. J Sci Food Agric.

[27] Hu Y, Pan Z, Huang Z, Li Y, Han N, Zhuang X, Peng H, Gao Q, Wang Q, Yang Lee BJ (2022). Gut microbiome-targeted modulations regulate metabolic profiles and alleviate altitude-related cardiac hypertrophy in rats. Microbiol Spectr.

[28] Zhang Q, Gao X, Wu J, Chen M (2022). The correlation between endotoxin, d-lactate, and diamine oxidase with endoscopic activity in inflammatory bowel disease. Dis Markers.

[29] Liu J, Yuan E, Zhang Z, Jia L, Yin Z, Meng X, Du H (2012). Age- and sex-specific reference intervals for blood copper, zinc, calcium, magnesium, iron, lead, and cadmium in infants and children. Clin Biochem.

[30] Geng ST, Zhang JB, Wang YX, Xu Y, Lu D, Zhang Z, Gao J, Wang KH, Kuang YQ (2021). Pre-digested protein enteral nutritional supplementation enhances recovery of CD4(+) T cells and repair of intestinal barrier in HIV-infected immunological non-responders. Front Immunol.

[31] Rzymski P, Tomczyk K, Rzymski P, Poniedzialek B, Opala T, Wilczak M (2015). Impact of heavy metals on the female reproductive system. Ann Agric Environ Med.

[32] Hertz-Picciotto I (2000). The evidence that lead increases the risk for spontaneous abortion. Am J Ind Med.

[33] Reckziegel P, Dias VT, Benvegnu DM, Boufleur N, Barcelos RCS, Segat HJ, Pase CS, Dos Santos CMM, Flores EMM, Burger ME (2016). Antioxidant protection of gallic acid against toxicity induced by Pb in blood, liver and kidney of rats. Toxicol Rep.

[34] Lafond J, Hamel A, Takser L, Vaillancourt C, Mergler D (2004). Low environmental contamination by lead in pregnant women: effect on calcium transfer in human placental syncytiotrophoblasts. J Toxicol Environ Health A.

[35] Holscher HD (2017). Dietary fiber and prebiotics and the gastrointestinal microbiota. Gut Microbes.

[36] Zheng D, Liao H, Chen S, Liu X, Mao C, Zhang C, Meng M, Wang Z, Wang Y, Jiang Q (2021). Elevated levels of circulating biomarkers related to leaky gut syndrome and bacterial translocation are associated with graves' disease. Front Endocrinol.

[37] Wan MLY, Ling KH, El-Nezami H, Wang MF (2019). Influence of functional food components on gut health. Crit Rev Food Sci Nutr.

[38] Myhrstad MCW, Tunsjo H, Charnock C, Telle-Hansen VH. Dietary fiber, gut microbiota, and metabolic regulation-current status in human randomized trials. Nutrients 2020. 10.3390/nu12030859. 10.3390/nu12030859 PMC7146107 32210176

[39] Cheng W, Lu J, Li B, Lin W, Zhang Z, Wei X, Sun C, Chi M, Bi W, Yang B (2017). Effect of functional oligosaccharides and ordinary dietary fiber on intestinal microbiota diversity. Front Microbiol.

[40] Sijbers AM, Schoemaker RJW, Nauta A, Alkema W (2020). Revealing new leads for the impact of galacto-oligosaccharides on gut commensals and gut health benefits through text mining. Benef Microbes.

[41] Krumbeck JA, Rasmussen HE, Hutkins RW, Clarke J, Shawron K, Keshavarzian A, Walter J (2018). Probiotic Bifidobacterium strains and galactooligosaccharides improve intestinal barrier function in obese adults but show no synergism when used together as synbiotics. Microbiome.

[42] Markowiak P, Slizewska K (2017). Effects of probiotics, prebiotics, and synbiotics on human health. Nutrients.

[43] Vandeputte D, Falony G, Vieira-Silva S, Wang J, Sailer M, Theis S, Verbeke K, Raes J (2017). Prebiotic inulin-type fructans induce specific changes in the human gut microbiota. Gut.

[44] Wang W, Chen D, Yu B, Huang Z, Mao X, Zheng P, Luo Y, Yu J, Luo J, Yan H, He J (2020). Effects of dietary inulin supplementation on growth performance, intestinal barrier integrity and microbial populations in weaned pigs. Br J Nutr.

[45] Awad WA, Ghareeb K, Passlack N, Zentek J (2013). Dietary inulin alters the intestinal absorptive and barrier function of piglet intestine after weaning. Res Vet Sci.

[46] Dall'Oglio F, Milani M, Micali G (2018). Effects of oral supplementation with FOS and GOS prebiotics in women with adult acne: the "SO Sweet" study: a proof-of-concept pilot trial. Clin Cosmet Investig Dermatol.

[47] Guo Q, Liu P, Zhou W, Xia M, Li J, Lu J, Ma JL, Chen ZJ, Yan J (2023). Growth hormone supplementation ameliorates blastocyst euploidy rates and improves pregnancy outcomes in women undergoing preimplantation genetic testing for aneuploidy cycles. Front Endocrinol.

